# Mixed Infection of Blackcurrant with a Novel Cytorhabdovirus and Black Currant-Associated Nucleorhabdovirus

**DOI:** 10.3390/v14112456

**Published:** 2022-11-06

**Authors:** Karel Petrzik, Jaroslava Přibylová, Josef Špak, Tatiana Sarkisova, Jana Fránová, Jan Holub, Jan Skalík, Igor Koloniuk

**Affiliations:** 1 Biology Centre Academy of Sciences of the Czech Republic, Institute of Plant Molecular Biology, 370 05 České Budějovice, Czech Republic; 2 Jan Holub Ltd., Hvozdečko 7, 783 25 Bouzov, Czech Republic

**Keywords:** aphid transmission, blackcurrant rhabdovirus 2, blackcurrant-associated rhabdovirus, electron microscopy, high throughput sequencing, *Ribes nigrum*

## Abstract

A virome screen was performed on a new breeding line, KB1, of blackcurrant. Rhabdovirus-like particles were observed by electron microscopy in ultrathin sections of flower stalks, and the complete genome sequence of a novel virus, provisionally named blackcurrant rhabdovirus 2 (BCRV2), was determined and verified using high-throughput sequencing. The genomic organization of BCRV2 was characteristic of cytorhabdoviruses (family *Rhabdoviridae*) and included seven genes: 3′-N-P′-P-P3-M-G-L-5′. BLASTP analysis revealed that the putative L protein had the highest amino acid sequence identity (75%) with strawberry virus 2. BCRV2 was detected in *Cryptomyzus galeopsidis*, but efficient transmission by this aphid was not confirmed. Of note, we observed coinfection of the KB1 line with blackcurrant-associated rhabdovirus (BCaRV) by RT-PCR. This is likely the first evidence of the presence of a cyto- and a nucleorhabdovirus in a single host.

## 1. Introduction

Plant-infecting rhabdoviruses are widespread and present in many dicotyledonous plants, including gymnosperms and monocotyledonous crops (barley, maize, rice, wheat) [1,2]. In addition, these viruses have been detected in less well-studied plants such as ferns and mosses, in liverworts [2], in ascomycetous fungi, and in lichens via RT-PCR or transcriptomics [3,4,5]. Many plant rhabdoviruses are transmitted by aphids, planthoppers, leafhoppers, whiteflies, and mites, in which they also replicate, so they may not be exclusively plant viruses. This shows that a negative-sense ssRNA genome with individually transcribed genes is functional in both plant and arthropod cells [6]. It has been hypothesized that plant-infecting rhabdoviruses evolved from ancestral arthropod viruses that gained the ability to infect plants as well [7].

To date, at least 20 viruses have been reported infecting *Ribes* plants [8]. Among them was the first observation of rhabdovirus-like particles in ultrathin sections of *Ribes* plants, with symptoms of blackcurrant reversion and gooseberry veinbanding diseases [9]. Later, rhabdovirus-like particles were observed in blackcurrant with symptoms of the severe form of blackcurrant reversion disease [10]. The particles were 200–347 nm by 64–90 nm in size. Blackcurrant-associated rhabdovirus (genus *Betanucleorhabdovirus*, BCaRV) was identified in the USA in 2018 in a single asymptomatic blackcurrant plant [11] and later documented in Latvia (Europe) [12]. Recently, Ribes virus 1 (RibV1) (*Varicosavirus*) was identified in the transcriptome of Siberian currant (*Ribes diacanthum*) [2]. The spread of the virus or its effects on the host, as well as its vectors, are not known. Some viruses, such as maize mosaic virus and strawberry crinkle virus, occur worldwide, but the spread of many other rhabdoviruses is limited and appears to be related to the distribution of insect vectors [6]. The other route of virus spread could be through infected propagules (shoots, buds) used for vegetative propagation of plant material.

The typical bullet-shape virus particles are easy to recognize, and electron microscopy has long been the main source of data on the presence of rhabdoviruses in plants. However, difficulties in mechanically transferring the virus to test plants and in isolating virus particles have resulted in incomplete biological information about many recognized rhabdoviruses, and there is a lack of sequence data for many of them.

The genomes of cyto- and nucleorhabdoviruses have six conserved canonical genes encoding nucleocapsid protein (N), phosphoprotein (P), putative movement protein (P3), matrix protein (M), glycoprotein (G), and large polymerase (L). Smaller additional genes may be located between the G and L genes or, in some viruses, between the N and P and/or the P and M genes [2,13]. Monocistronic, 3′-polyadenylated, 5′-capped, positive-sense RNAs are transcribed from the genome. A conserved gene junction, which is important for gene transcription and replication, separates each gene. The dense core of rhabdoviral particles, as seen in ultrathin sections using an electron microscope, consists of the genomic RNA, which is tightly associated with the N protein and the P, M, and L proteins. The glycoprotein (G) forms the glycoprotein spikes of the rhabdovirus virions which presumably interact with receptors in the vector midgut [14].

Currants are propagated vegetatively, but new varieties are produced by sexual crossing [15]. Crossing is also a route by which seed-borne viruses are transmitted to the new hybrid. Vertical transmission of plant viruses can occur via seed, but this is not very common, because most viruses do not enter the embryo [16]. Varicosaviruses (bi-segmented rhabdoviruses) might be vertically transmitted [2], although seed transmission of rhabdoviruses has not been elucidated. Notably, Suaeda salsa cytorhabdovirus has been identified, and its nearly complete genome sequence has been reconstructed from transcriptome data of *Suaeda salsa* seeds [1,17] as well as RibV1. Finally, Rudbeckia virus 1 cytorhabdovirus has been detected in rudbeckia seeds [18], so transmission of some rhabdoviruses through seeds cannot be excluded.

In this work, we describe, for the first time, a mixed infection of blackcurrant with a novel cytorhabdovirus and blackcurrant-associated rhabdovirus, present a genome description of the former virus, and explore potential vectors and sources of the novel virus.

## 2. Materials and Methods

Plants of the new KB1 breeding line of a dessert variety of blackcurrant were provided by Holub Ltd., Czech Republic. The material showed no visible mosaic, growth malformation, or other symptoms indicating the presence of intracellular pathogens.

Ultrathin cross sections were prepared from the flower stalks from three greenhouse-grown KB1 plants at the flowering stage. Tissue pieces (approx. 2 mm × 2 mm) were fixed in 0.1 M potassium phosphate buffer, pH 7.3, 5% glutaraldehyde and 4% sucrose for 24 h at 4 °C under light vacuum. Samples were post-fixed in 1% osmium tetroxide, dehydrated in ethanol and embedded in Durcupan resin (Fluka). Ultrathin sections 50 to 70 nm thin were stained with uranyl acetate and examined in a TEM JEOL 1400 at 120 kV.

Total RNA was purified from approximately 50 mg of leaves using the GeneJET Plant RNA purification mini kit (Thermo Fisher Scientific, Waltham, MA, United States) and used for library preparation with the NEBNext Ultra II Directional RNA Library Prep Kit and Illumina sequencing with the NEBNext Poly(A) mRNA Magnetic Isolation Module (both NEB, Ipswich, MA, United States). Batches of 10 seeds were surface sterilized with 5% perchlorate for 15 minutes, washed three times with distilled water and used for RNA purification. Total RNA was purified from individual aphids using TRI reagent (Sigma Aldrich, St. Louis, MO, USA) according to manufacturer’s recommendation. Molecular identification of aphid species was based on Sanger sequencing of the *cox b* gene with published primers [19] and Phire Tissue Direct PCR Master Mix according to the manufacturer’s recommendation (Thermo Scientific, Waltham, MA, USA).

RT-PCR and RT-qPCR analyses were performed using cDNA templates prepared using RevertAid transcription kit (Thermo Scientific, Vilnius, Lithuania) and SapphireAmp PCR mastermix (Takara Bio, Shiga, Japan) and HOT FIREPol EvaGreen qPCR Mix Plus (Solis BioDyne, Tartu, Estonia), respectively. Detection primers and primers used for genome ends sequencing are presented in Supplementary Table S1. The amplification reactions were performed with 35 cycles of 95 °C for 5 s, 60 °C for 20 s, and 72 °C for 10 s. 

High-throughput sequence data were analysed in CLC Genomic Workbench 9.1.5 (Qiagen, Hilden, Germany), and general sequence analyses were performed in Geneious 9.1.8 (Biomatters, New Zealand). The 5′ and 3′ends were obtained using the 5′ and 3′ RACE kits (Thermo Scientific, Invitrogen, Waltham, MA, United States) and the specific primers listed in Supplementary Table S1.

Phylogenetic analysis on N and L proteins was performed using MEGA X software with the ML method and the JTT model [20] and 1000 replicates.

N-glycosylation sites in the G and P′ proteins were predicted at https://services.healthtech.dtu.dk/service.php?NetNGlyc-1.0. accessed on 1 July 2022.

For aphid-transmission experiments, three KB1 leaves with natural populations of *Aphis shubertii* and *Cryptomyzus galeopsidis* (approximately 100 individuals) were placed together in net-protected cages containing BCRV2- and BCaRV-free blackcurrant cv. Titania and cv. Öjebyn plants for three weeks. Prior to transmission assays, individual aphids from KB1 plants were RT-PCR tested with BCRV2-specific primers 3395 and 3396 and BCaRV-specific primers 3136 and 3137 (Supplementary Table S1).

Relative virus titer was estimated using NADH mRNA as a reference [21] with three technical replicates. Data analyses were performed using Bio-Rad CFX Maestro 1.1, version 4.1 (Bio-Rad), R version 4.1.0 (8 May 2021) [22] and the ggplot2 package version 3.3.5 [23] under RStudio version 2021.09.1+372.

## 3. Results

### 3.1. Morphology and Localization

**Figure 1:**
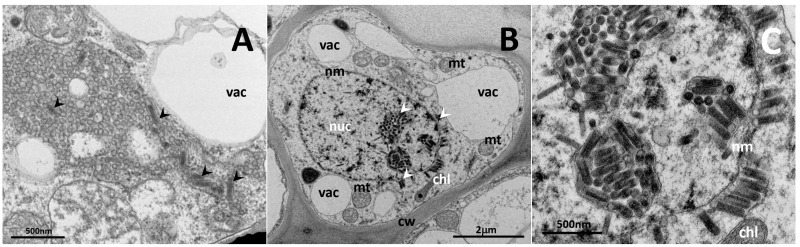
Rhabdovirus-like particles (marked with arrowheads) observed in ultrathin-sections of companion cells of blackcurrant flower stalks. (**A**) Rhabdovirus-like particles in the cytosol. (**B**) Rhabdovirus particles in the nucleus and perinuclear space. (**C**) Detail of rhabdovirus particles in the nucleus. nuc-nucleus, nm-nuclear membrane, vac-vacuole, cw-cell wall, chl-chloroplast, mt-mitochondrion.

In all three preparations of blackcurrant plants of the KB1 cultivar line, rhabdovirus-like particles were found in ultrathin cross sections of flower stalks by electron microscopy. The particles occurred in membrane-bound aggregates within nuclei, as well as in the cytoplasm of parenchyma cells of vascular bundles and in companion cells of vascular bundles. The putative nucleorhabdovirus particles were 76 ± 6.6 × 253 ± 15.8 nm in size (*n* = 19), whereas the cytorhabdovirus particles were smaller, measuring 61 ± 4.3 × 225 ± 21.4 nm (*n* = 11) (Figure 1A–C).

### 3.2. Genome Organisation

Two nearly complete genome sequences were assembled from the HTS data. The first sequence, 14.364 nucleotides (nt) long, was 89.9% identical at the nt level to blackcurrant-associated rhabdovirus (BCaRV) [11].

**Figure 2:**
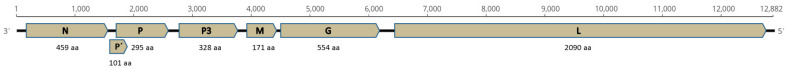
Genome arrangement of the blackcurrant rhabdovirus 2 (BCRV2). All open reading frames are in scale.

**Figure 3:**
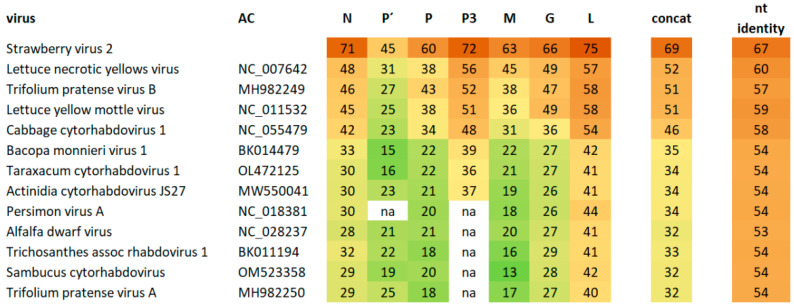
Shared percent amino acid sequence identity of BCRV2 in distinct proteins, concatenated proteins, and genome (nt) identity with related cytorhabdoviruses. Na-not applicable.

The second viral genome was assembled from 13,758 individual reads, resulting in a mean genome coverage of 159. This represents approximately 0.02% of the 81 million trimmed reads. The 3′ and 5′ ends were completed using the RACE approach. The complete genome of blackcurrant cytorhabdovirus is 12,882 nt in length. This is one of the smallest genomes among cytorhabdoviruses (the smallest genome has 12,193 nt, the largest 14,961 nt). The genome termini are complementary in a 24 nt long region, with 1 nt long overhang at the 3′ terminus. The genome contains seven genes in the order 3′-N-P′-P-P3-M-G-L-5′ (Figure 2). The 3′-leader sequence is relatively short compared with related viruses at 161 nt, as is the 5′-trailer sequence at 183 nt. Strawberry virus 2 (StrV2) proteins are the most similar to BCRV2, with amino acid sequence identities between 45 and 75% for different genes. Lettuce necrotic yellows virus (LNYV) proteins are the second most similar, with sequence identities between 31 and 57% (Figure 3). Four transmembrane motifs were detected on P’ protein, and two transmembrane motifs were detected on the encoded G protein. Three N-glycosylation sites were also detected in the G protein. Identities of 69.4% and 67% between the newly sequenced cytorhabdovirus and StrV2 are below the thresholds of current species demarcation criteria for cytorhabdoviruses (less than 75% nucleotide sequence identity of the complete genomes and less than 80% amino acid sequence identity in all cognate reading frames), respectively [24]. Based on this finding, we propose that the novel virus, tentatively named blackcurrant rhabdovirus 2 (BCRV2) be classified as a new species *Cytorhabdovirus ribesnigri* in the genus *Cytorhabdovirus*, family *Rhabdoviridae*. 

**Figure 4:**
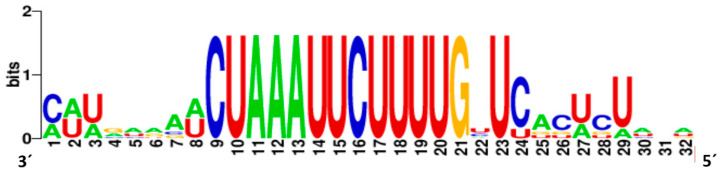
Weblogo of the conserved junction sequence of the BCRV2 (viral genomic strand is shown).

The conserved gene junction sequences that have been shown to play an important role in transcription termination and polyadenylation of the preceding gene transcript and transcription initiation of the following transcript are 3′ AAUUCUUUU, GNU, and C(N) (Figure 4). This motif is very similar to that of StrV2, LNYV, lettuce yellow mottle virus, Trifolium pratense virus B, and cabbage cytorhabdovirus 1 [1,25].

### 3.3. Phylogeny 

**Figure 5:**
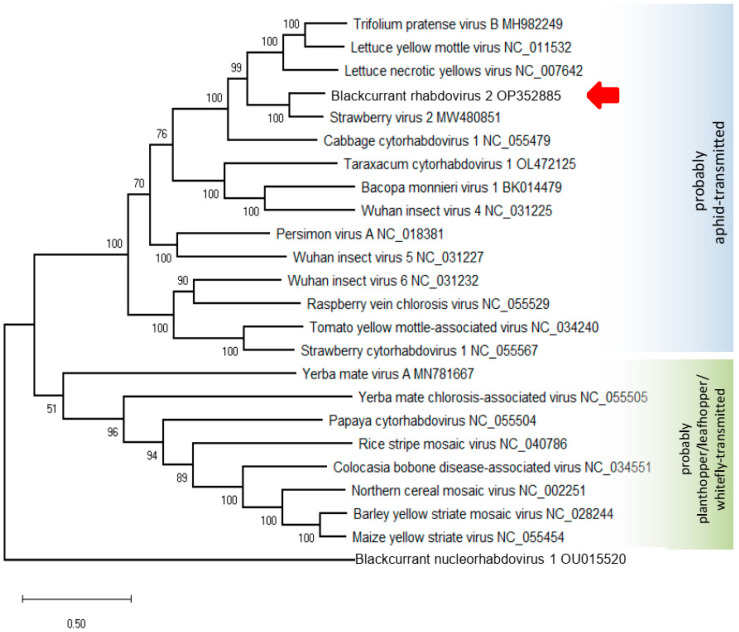
Maximum likelihood phylogenetic tree inferred from the amino acid sequences of the L protein of selected cytorhabdoviruses. Bootstrap = 1000 replicates.

The phylogenetic trees based on the L and N protein amino acid (aa) sequences are topologically identical and show that BCRV2 clustered with StrV2, LNYV, Trifolium pratense virus B, and cabbage cytorhabdovirus 1, among the cytorhabdoviruses (Figure 5).

### 3.4. Aphid Vectoring 

In plant rhabdoviruses, there is a strong correlation between phylogenetic relationships and vector types [6]. Phylogenetic clustering of BCRV2 among aphid-borne viruses leads to the hypothesis that aphids are the natural vectors of this virus. Moreover, the accessory P’ protein is encoded by all cytorhabdoviruses that appear to be transmitted by aphids [1]. 

Two plants of the KB1 breeding line, kept separately in a greenhouse, were naturally colonized by two aphid species: the European blackcurrant aphid (KB1-1 plant, *Cryptomyzus galeopsidis*) and the permanent currant aphid (KB1-2 plant, *Aphis schneideri*). Aphids from both species were screened for the presence of cyto- and rhabdoviruses using virus-specific primers and RT-PCR. *A. schneideri* aphids (*n* = 8) were negative for both viruses, while five *C. galeopsidis* aphids were positive for BCRV2 and two were positive for BCaRV. However, the transmission experiments with *C. galeopsidis* (approximately 100 aphids transmitted per plant) did not result in BCRV2 infection of cv. Titania (*n* = 3) or cv. Öjebyn (*n* = 2) plants, when tested by RT-PCR 17- and 28-days post aphid transmission. 

### 3.5. Relative Titers of the Cytorhabdovirus and the Nucleorhabdovirus in a Single Plant

**Figure 6:**
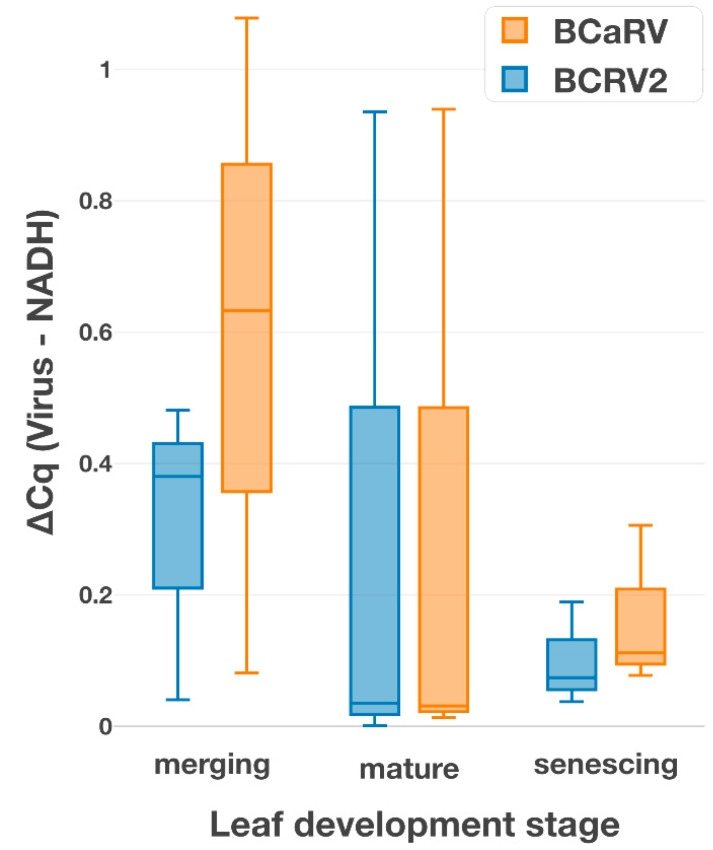
Comparison of relative N gene expression of BCRV2 and BCaRV in leaves of different development stage with the NADH expression.

Mixed infection with two rhabdoviruses (one cyto- and one nucleorhabdovirus) in a single plant raises new questions about possible competition between the viruses. Therefore, we determined the relative titers of both viruses using NADH mRNA as a housekeeping reference. Although we detected higher BCRV2 levels than BCaRV in individual KB 1 plants using the RT-qPCR-based analyses, the differences were not statistically significant and varied widely among biological samples (*n* = 3). Similarly, only minor differences (up to Cq = 1) were detected between leaves at different developmental stages (young, adult, mature) with the same result (Figure 6). 

**Figure 7:**
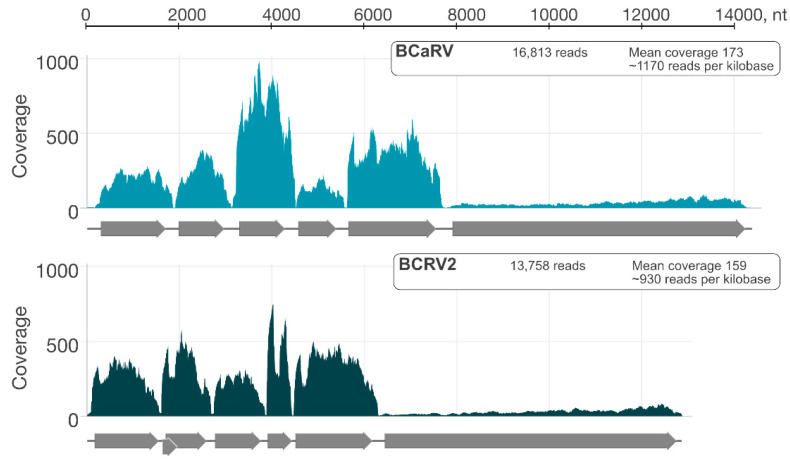
High-throughput sequence coverage of BCaRV and BCRV2.

These results confirm the findings of HTS, where the sequence coverage of the BCaRV genome was almost identical to that of BCRV2 (Figure 7). It can be concluded that there is no significant difference in the level of accumulation between the two viruses. 

### 3.6. Origin of BCaRV and BCRV2

There were no specific symptoms on the blackcurrant plants that indicated the presence of rhabdoviruses. We therefore collected representative samples from two breeding lines, KB1 and KB2, and samples from several of the breeder’s cultivars and from other growers and locations in order to investigate virus incidence. Eleven of fourteen samples were positive for BCaRV in RT-PCR, and six samples were positive for both BCaRV and BCRV2. The N gene of the two BCRV2 positive isolates was sequenced, and the sequences were found to be identical to the sequence assembled from HTS. This indicated low genetic diversity and dissemination of the virus via propagation material. In addition, BCaRV was detected in one of 16 samples from other sources, but BCRV2 was not detected outside the original site. Based on these results, we hypothesized that the source of BCaRV was virus-infected parent plants used for breeding. Notwithstanding the limited sample size, this hypothesis was supported by the occurrence of the virus in germplasm collections [12]. However, this route of infection requires transmission of the virus by pollen or seed, which has not yet been confirmed. Therefore, the origin of BCRV2 remains unknown. It is probably a relatively recent infection, occurring only in the new breeding line KB1/KB2 and not in other cultivars or in vitro cultures.

## 4. Discussion

Although high-throughput sequencing is now a relatively accessible, sensitive, and inexpensive method for virus screening, it requires some skill, expertise, and specialized software that are not routinely available in every laboratory. Using the simpler RT-PCR and degenerate primers that recognize the most conserved motifs on the L gene of rhabdoviruses, we detected cytorhabdoviruses in ivy plants and Usnea lichens [3,4]. Using the same primers, we obtained a specific RT-PCR product from randomly selected red currant plants, i.e., cv. Holandský červený. The nucleotide sequence of this conserved part of the L gene is highly identical (99.4%) to the recently described Trifolium pratense virus A (genus *Cytorhabdovirus*) [13], so this virus is probably an isolate of Trifolium pratense virus A that can infect currants. This is a small comment on the fact that, so far, only three rhabdoviruses have been described in currants, and the host range of the different rhabdoviruses is not known at all. 

Blackcurrant-associated rhabdovirus (BCaRV) was previously identified in the United States in blackcurrant germplasm imported from Russia, accession Veloy [11]. BCaRV was also found in cv. Burga from France [11]. Zrelows et al. [12] documented the natural occurrence of BCaRV in the asymptomatic blackcurrant cv. Mara Eglite in Latvia, which was the second case of BCaRV occurrence in Europe. Together with our findings of BCaRV in the KB1 and KB2 lines, in cvs. Kopania, Noiroma in the Czech Republic, BCaRV appears to be widespread in blackcurrants in Europe.

The sizes of the particles of both rhabdoviruses observed in ultrathin sections were very similar. This prevented us from accurately identifying the nature of individual particles located outside the nucleus. In this case, specific labeling should be used for such differentiation. 

Although *Ribes* species harbor more than 30 aphid species [26,27], among which *Aphis schneideri, C. galeopsidis, C. ribis, Hyperomyzus lactucae*, and *Nasonovia ribisnigri* are the most common, we found only two of them on the blackcurrant plants selected in this study. Although some *C. galeopsidis* individuals were positive for both viruses, virus transmission was not successful with a larger number of these aphids. It should be noted that BCRV2 is phylogenetically related to cytorhabdoviruses, whose vectors are aphids. Of particular interest would be other aphid species native to *Ribes* sp. The G protein of BCRV2, which is essential for the attachment of the virus to predicated midgut receptors of aphid vectors [14], is very similar to that of StrV2 (66%) (Figure 3). Perhaps we need to investigate strawberry-sucking aphids for the natural aphid vector of BCRV2. 

In mixed virus infections, there are different types of relationships between virus species, ranging from antagonism to mutualism [28]. We found that BCaRV and BCRV2 had similar titers in the co-infected blackcurrant leaves tested in this study. However, it would be of particular interest to compare virus titers in blackcurrant with single and double infections to determine the relationship between these two viruses. 

The effects of the viruses on blackcurrant yield are not yet known. It is somewhat surprising that plants infected with the two viruses (resulting in asymptomatic infection) have been recognized as promising for further evaluation, with good flavor and fruit size characteristics. We note that infection with a similar aphid-borne cytorhabdovirus, strawberry virus 1, reduces yield per plant by 11.7–31.8%, depending on the cultivar [29].

Further research should focus on identifying the natural BCRV2 vector by increasing the number of transmission experiments, and determining the biological significance of the virus in terms of its incidence and impact on yield.

## 5. Conclusions

- To our knowledge, this is the first documented case of mixed cyto- and nucleorhabdoviral infection in a single host.
- Interestingly, *Aphis schneideri* seems to be unable to replicate and transmit BCRV2 and BCaRV. While part of the population of *Cryptomyzus galeopsidis* was positive for both viruses, it was unable to transmit the viruses to two blackcurrant cultivars.

## Data Availability

The complete nucleotide sequence of the blackcurrant rhabdovirus 2 (BCRV2) and blackcurrant nucleorhabdovirus are deposited in GenBank under the accession numbers OP352885 and OP352886, respectively. The partial sequence of the L gene of red currant Trifolium pratense virus A has GenBank accession number OP329723. The *Cox* gene sequences of *A. schneideri* and *C. galeopsidis* have GenBank accession numbers OP375537 and OP355325, respectively.

## Supplementary Materials

The following supporting information can be downloaded at: https://www.mdpi.com/article/10.3390/v14112456/s1; Table S1: Primers used in this work.
