# Supplementary material for: Mixed Infection of Blackcurrant with a Novel Cytorhabdovirus and Black Currant-Associated Nucleorhabdovirus
Source: Viruses. 2022 Nov 6;14(11):2456. doi: 10.3390/v14112456 (PMC9697673; doi:10.3390/v14112456)
Supplement: Supplementary file 1 [file viruses-14-02456-s001.zip › viruses-1931462-supplementary.pdf]

viruses

MDPI

Supplementary Material

Supplementary Table S1. Primers used in this work.

primer	5′- 3′	use
3191	CCTCTGCAACCTTTCTTGGA	3′RACE
3192	GCCCAAACTTCTGAGGTTCT	3′RACE
3193	ACCATTGCCTCTCCTAAAGC	3′RACE
3197	CCCACATCTTACAAGCCGAT	5′RACE
3198	CGGCATCTTTGAAAGCAGAC	5′RACE
3199	CCAGTGCGATCTCCTGATTT	5′RACE
3136	CCATGTTCAGGACATTCAAACC	RT-qPCR BCaRV
3137	ACATTGAAGATTTTTGGACTAGGTTTA	RT-qPCR BCaRV
3395	CACAGGGGAAGTGTCATACC	RT-qPCR BCCRhV1
3396	TACAGTTAGCTCCCGATCGA	RT-qPCR BCCRhV1
AtropaNad2.1a	GGACTCCTGACGTATACGAAGGATC	RT-qPCR NADH
AtropaNad2.2b	AGCAATGAGATTCCCCAATATCAT	RT-qPCR NADH
LV	GAGIACYTGRTTRTCICC	Rhabdo-specific
DF	GAYTTYGARAARTGGAACGG	Rhabdo-specific
